# Thermal Diffusivity Characteristics of the IN718 Alloy Tested with the Modified Pulse Method

**DOI:** 10.3390/ma15227881

**Published:** 2022-11-08

**Authors:** Janusz Terpiłowski, Stanisław Jóźwiak, Grzegorz Woroniak, Robert Szczepaniak

**Affiliations:** 1 Faculty of Mechatronics, Armament and Aerospace, Military University of Technology, Gen. S. Kaliskiego Street No 2, 00-908 Warsaw, Poland; 2 Institute of Materials Science and Engineering, Military University of Technology, Gen. S. Kaliskiego Street No 2, 00-908 Warsaw, Poland; 3 HVAC Department, Bialystok University of Technology, 15-351 Bialystok, Poland; 4 Faculty of Aviation, Polish Air Force University, Dywizjonu 303 Street No 35, 08-521 Deblin, Poland

**Keywords:** thermal diffusivity, modified pulse method (MPM), alloy 718, flash method

## Abstract

The article presents the use of the modified pulse method (MPM) to determine the temperature characteristics of the thermal diffusivity of alloy 718. The experiment was carried out in the temperature range of 20–900 °C during the double heating of the sample with an interval of two weeks. The results of our own research showed a good correlation in the temperature range of 300–500 °C, during the first heating of the sample, with the recommended changes in thermal diffusivity by NPL & ASM and data from the MPDB database. On the other hand, clear deviations in the results occurred in the range of temperature changes up to about 300 °C, most likely responsible for the electron component of the conductivity of this alloy, and in the range above 700 °C, where there is a clear minimum that may be caused by the δ phase precipitation phenomenon.

## 1. Introduction

Thermal diffusivity is an important thermophysical property because it is suitable for predicting material behavior in many heat transfer applications and plays an important role in materials science. A short review of the methods for thermal diffusivity determination such as the laser flash method, single side flash method, thermal wave interferometry (TWI), etc. can be found in [1,2,3,4,5,6,7,8,9,10,11]. Since the introduction of the thermal flash technique, it has become a leading method for measuring the thermal diffusivity of solids. Reza et al. [12] and Bellucci et al. [13] used this parameter in their investigations connected with deuterium implanted tungsten and in their research on graphene nano-platelets. This work shows ways of assessing the thermophysical properties of a material using a parameter (i.e., thermal diffusivity by means of the modified flash method (MPM)). It seems that the use of this method, significantly different from other methods used to determine the thermal diffusivity of solids [10,14,15,16,17,18,19,20,21,22,23], is justified mostly by its much higher accuracy in determining the a(T) of the tested materials.

The authors first work in this area [24] concerned the research and interpretation of the temperature characteristics of the *a*(T) Fe61Ni39, Fe52Ni48, and Fe40Ni60 alloys, with the temperature range of 20–700 °C. Another work [25] concerned the research and interpretation of the properties of the *a*(T) metastable Fe80Ni20 alloy, with the temperature range from ambient to about 650 °C. This work concerns the research and interpretation of the thermal diffusivity characteristics $a\left(T\right)$ of the Inconel 718 superalloy to a temperature close to 900 °C.

Inconel 718, an austenitic high-temperature Ni–Cr–Fe alloy, is one of the most commonly used materials in the family of nickel-based superalloys. It maintains the face-centered cubic (fcc) crystal structure from an ambient temperature to a high melting point temperature around 1300 °C [26,27]. It is characterized by an improved balance of high strength, creep, and tensile properties as well as excellent corrosion and oxidation resistance at elevated temperatures up to 700 °C, which is why they are attractive as construction materials for numerous applications at high temperatures (including elements in rockets, rings, aircraft and turbine engines running on liquid fuel) [28]. However, in recent years, there has been an increasing demand for high-alloy nickel-based superalloys to meet the requirement of a higher speed and thrust-to-weight ratio for advanced aircraft engines [29]. This is a really big challenge for the Inconel 718 superalloy, which hinders its further application in the aerospace industry. Due to this demand, attempts are being made to improve the properties of alloy 718 by adding some additives [30] or by changing the production method. Therefore, the additive manufacturing method is becoming extremely popular [31,32,33,34,35,36,37,38]. All changes and modifications in the area of chemical composition and the manufacturing technology affect the precipitation phenomena that determine the structure of the alloy. Classically used for operation at temperatures up to 700 °C, Inconel 718 [39] is a superalloy with a solid solution matrix fcc-γ, strengthened with plate precipitates of the tetragonal phase γ″ (Ni_3_Nb, bct_D022) and the cubic superstructure γ′ (Ni_3_(Al, Ti, Nb), fcc_L12) and carbides, mainly NbC [40]. Moreover, at high temperatures, harmful δ (Ni_3_Nb, D0a) and Laves ((Ni, Fe, Cr)_2_ (Nb, Ti, Mo)-hexagonal C14) phases may form in the alloy structure. Interestingly, the precipitation processes of the harmful phase δ can take place within the alloy grains already at the temperature of 700 °C after 10^−3^ h [40], affecting the changes in the properties including the thermal properties of the material.

Therefore, it seems necessary to test the alloy after each applied structural and technological modification, not only in terms of the mechanical properties, but also thermophysical properties such as thermal diffusivity, which can be tested with a fairly accurate method such as MPM. The MPM for measuring thermal diffusivity has been described in detail in [41,42,43].

## 2. Experiments

The determination of thermal diffusivity by means of the MPM is based on the theoretical determination of the temperature distribution inside an opaque and adiabatic sample as well as the difference in temperature between two opposite surfaces after the laser pulse is fired on its front surface. In this case, a one-dimensional model is assumed, which approximates the actual heat exchange in the “sample–environment” system. The next step in the research is to record a temporary temperature difference between the front and back surfaces of the sample, resulting in a one-dimensional process of temperature equalization in the sample. Finally, we estimated how to match the results of the experiment with a curve in the best way, obtaining them as one of several theoretical curves that solve the problem. The optimization parameter is thermal diffusivity and the value corresponding to the best match is considered a proper one.

**Figure 1:**
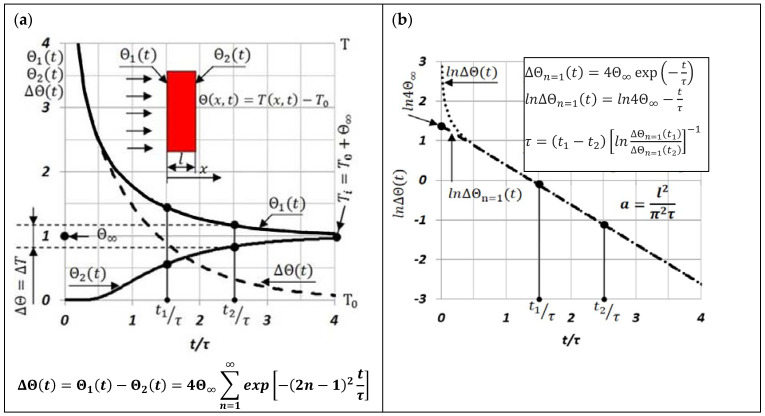
The rule for determining the thermal diffusivity of a sample using the MPM: (**a**) temperature changes on opposite surfaces of the sample and its difference; (**b**) procedure for determining the characteristic time τ and thermal diffusivity a(T) of the sample.

The rule for determining the thermal diffusivity *a*(T) of a sample using the MPM is presented in Figure 1.

**Figure 2:**
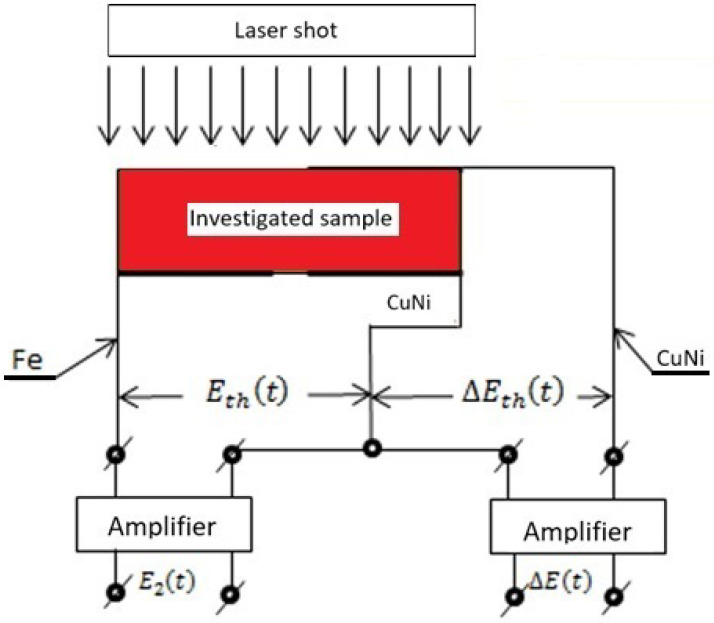
The measuring system enables simultaneous measurement of the temperature difference ∆E(t) on the extreme surfaces of the sample and on its back surface E_2_(t) after the laser shot.

Figure 2 shows a practical way to determine the temperature difference $\Delta \Theta \left(t\right)$ and the temperature $\Theta_{2}\left(t\right)$ on the back surface of the test sample from Inconel 718, if thermocouple sensors are used to measure them. In this case, it was assumed that:

- The Seebeck coefficient $k_{1}$ of the differential thermocouple “CuNi–sample–CuNi” is determined from the dependence $\Delta E\left(t\right)=Kk_{1}\Delta \Theta \left(t\right)$;
- The Seebeck coefficient $k_{2}$ of the thermocouple “Fe–CuNi” is determined from the dependence $E_{2}\left(t\right)=Kk_{2}\Theta_{2}\left(t\right)$;
- If the variations of $\Delta \Theta \left(t\right)$ and $\Theta_{2}\left(t\right)$ are minor, then $k_{1}$ and $k_{2}$ are constant values;
- The gain factor of both amplifiers was constant and amounted to $K=10^{4}\;V/V$.

Then, the dependence on $\Delta \Theta \left(t\right)$, shown in Figure 1, assuming that the changes in this difference are small, can be written for the purposes of the experiment (Figure 2) in the form:

(1) $$\Delta E_{th}\left(t\right)=4E_{\infty }\overset{\infty }{\underset{n=1}{\sum }}exp\left[-{\left(2n-1\right)}^{2}\frac{t}{\tau }\right]$$

where:

(2) $$\tau =\left(t_{2}-t_{1}\right){\left[ln\frac{\Delta E_{th}\left(t_{1}\right)}{\Delta E_{th}\left(t_{2}\right)}\right]}^{-1}$$

Since the Seebeck coefficient $k_{1}$ of the tested material is usually unknown, and its value is necessary to determine the temperature increase $\Theta_{\infty }$ of the tested sample after a laser shot at its front surface, the following procedure was used to determine it. On one hand, the thermocouple used to determine the temperature rise on the back surface of the sample $\Theta_{2}\left(t\to \infty \right)\;$= $\Theta_{\infty }$ should be selected so that its temperature characteristics are known $E_{th}\left(t\right)=k_{2}\Theta_{2}\left(t\right)$, and hence the ability to specify the value of $\Theta_{\infty }$. On the other hand, in the course of the same experiment, the characteristic time τ and the values $E_{\infty }=0.25exp\left[ln\Delta E_{th,n=1}\left(t=0\right)\right]$, were determined simultaneously from the parallel registered changes $\Delta E\left(t\right)$, as shown in Figure 2.

The known and experimentally determined values of $\Theta_{\infty }$ and $E_{\infty }$ allow for determining the sought value of the Seebeck coefficient $k_{1}$ of the “Ni–CuNi” thermocouple, from the dependence $E_{\infty }=k_{1}\Theta_{\infty }$.

## 3. Measurements

**Figure 3:**
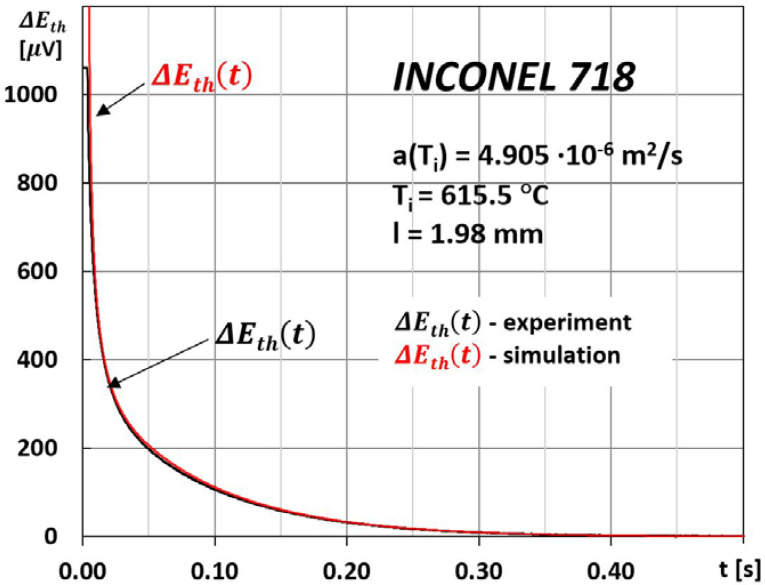
Recorded changes in the thermoelectric voltage $\Delta E_{th}\left(t\right)$ between the extreme surfaces of the sample after a laser shot at its front surface (Figure 1 and Figure 2) and their simulation based on the determined values of τ and $E_{\infty }$ (Figure 4).

The measure of the correctness of the $E_{\infty }$ and the characteristic time τ values determined by this method, and thus the thermal diffusivity $a(T_{i})$, are the result of comparing the changes $\Delta E_{th}\left(t\right)$ from the experiment with their simulation, as shown in Figure 3.

**Figure 4:**
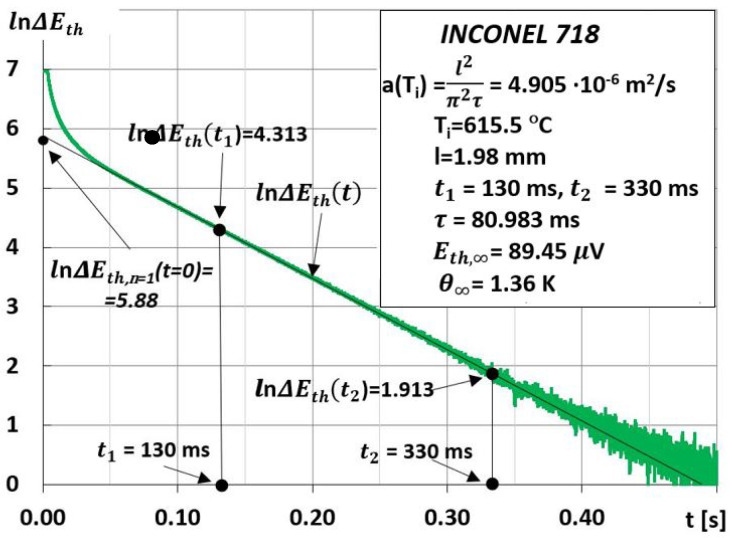
The method of determining the characteristic time τ and thermal diffusivity $a\left(T_{i}=T_{0}+\Theta_{\infty }\right)=l^{2}/\left(\pi^{2}\tau \right)$ at the temperature $T_{i}$ based on the course of changes in the function $y=ln\left[\Delta E_{th}\left(t\right)\right]$, where $\Delta E_{th}\left(t\right)$ is shown in Figure 3.

The time interval between subsequent discrete measurements of $a(T_{i})$ was dictated by the establishment of heat exchange conditions in the tested sample and was equal to approximately 20 min. An exemplary method of developing the result of the measurement of thermal diffusivity at a discrete temperature T_0_ of sample thermostating using the MPM method is shown in Figure 3 and Figure 4.

**Figure 5:**
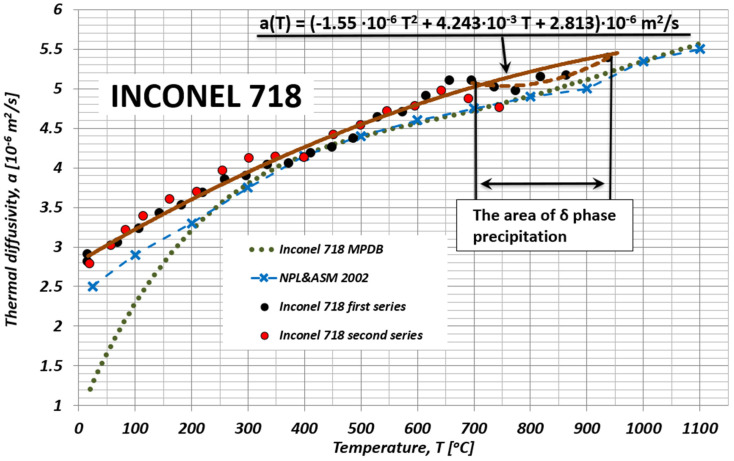
Temperature characteristics of the IN718 alloy’s thermal diffusivity and its approximation in comparison with the results from the MPDB database [45] and the NPL results [44].

However, Table 1 and Figure 5 present the authors’ own results of the IN718 alloy’s chemical compositions and thermal diffusivity investigations, together with the results published by the NPL & ASM [44] and by the MPDB database [45].

**Figure 6:**
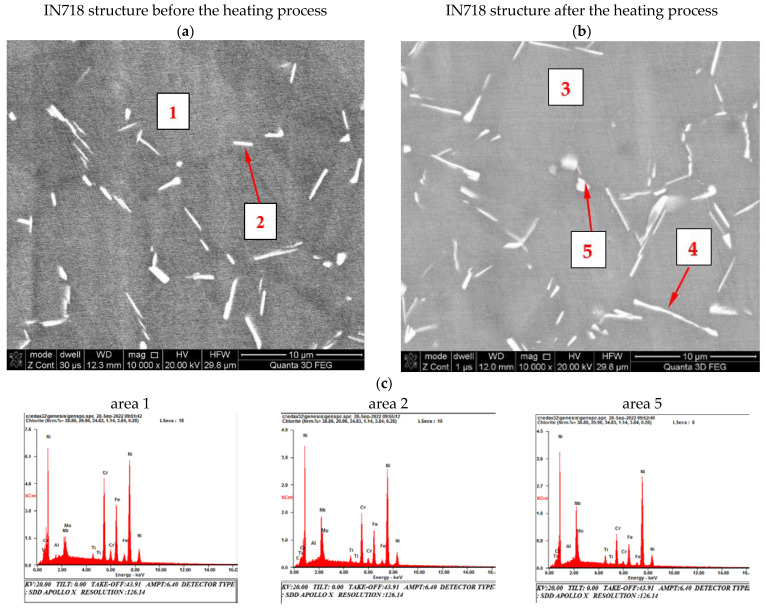
The structure of the solid γ solution with precipitates within the grain boundaries of the IN718 alloy before the heating process (**a**) and after the heating process (**b**) with exemplary EDS spectra (see Table 2) from the marked areas (**c**).

**Figure 7:**
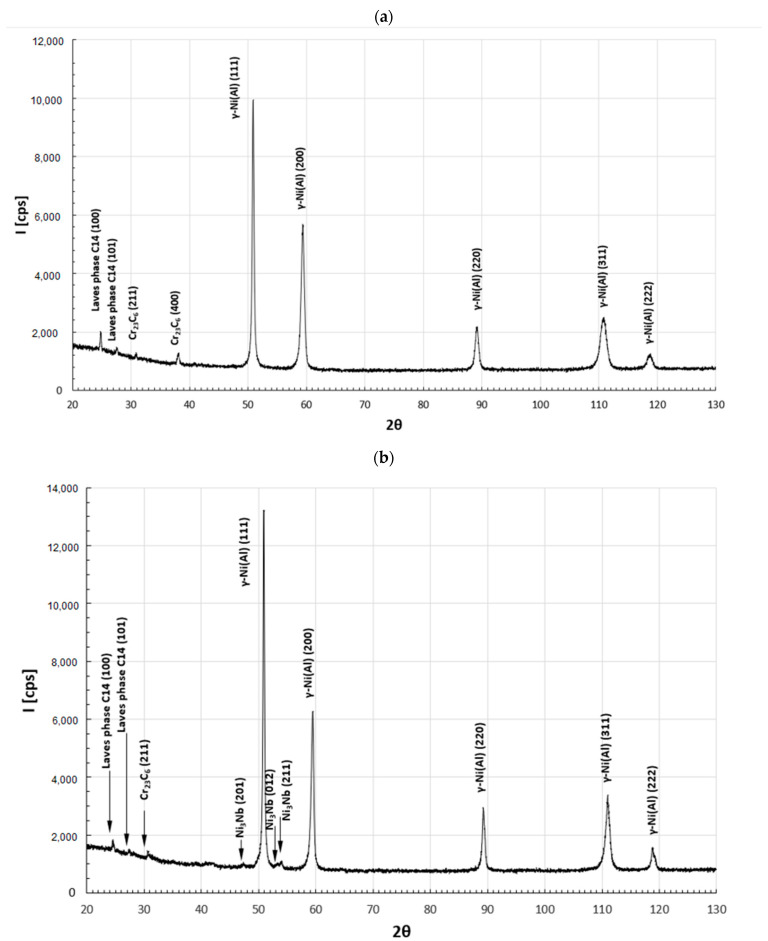
XRD patterns obtained for the IN718 alloy before the heating process (**a**) and after the heating process (**b**).

The thermal diffusivity of the Inconel 718 alloy by the modified MPM pulse method was tested twice, with an interval of two weeks during the heating cycle, on the one and the same 1.98 mm thick sample. The tests were carried out in the range of temperature changes from room temperature to approximately 1000 °C. In order to explain the non-monotonic change in diffusivity, observed in the temperature range of 700–1000 °C, the recorded course was correlated with the structural changes of the tested material. This analysis was performed based on the microstructural SEM observations supported by the EDS microanalysis of the chemical composition and the X-ray phase XRD analysis (Figure 6 and Figure 7).

The alloy structures were observed on the Quanta 3D FEG field emission scanning electron microscope (SEM) (Field Electron and Ion Company, FEI, Hillsboro, OR, USA), which allowed us to first perform a chemical composition analysis using an energy-dispersive X-ray spectroscopy (EDS) detector and an additional microdiffraction using an electron backscatter diffraction detector (EBSD). The phase fractions in the material were determined by using X-ray diffraction (XRD) analysis on a Rigaku X-Ray Diffractometer Ultima IV (Rigaku, Tokyo, Japan) with a Co lamp (λ = 1.79 Å) and PDF-4 database.

## 4. Discussion

The results of our own research showed a good correlation in the temperature range of 300–500 °C, during the first heating of the sample, with the recommended changes in thermal diffusivity by NPL & ASM [44] and data from the MPDB database [45], on the basis of which, using the relation $a\left(T\right)=\lambda /\left(\rho c_{P}\right),$ (where: λ—thermal conductivity, ρ—density, $c_{P}$—specific heat), it was possible to determine the thermal diffusivity.

It seems that the difference between the values from the authors’ own research a(T) as well as those published in Milles’s monograph [44] and in the MPDB database [45] in the range of temperature changes up to 300 °C was caused by the failure to take into account the contribution of the electron component during the registration of temperature changes at the back surface of the test sample after a laser shot at its frontal surface. This is most likely due to the fact that the surfaces of the samples prepared for testing a(T) are covered with a thin layer of colloidal graphite. Then, from the frontal side, it shields the subsurface layer of metal against a direct interaction of the laser pulse with the conduction electrons. Thus, the one-dimensional process of heat conduction inside the sample can be considered as equilibrium. Additionally, in the currently used thermal diffusivity measuring devices, temperature detectors are sensors that are sensitive to near and medium infrared, unlike panchromatic detectors (thermocouples).

An equally important reason for the discrepancy between the results of the authors’ own research a(T), and those published in other sources, may be the thermal history of the tested sample, before and during the experiment, which is shown in Figure 5. In the research presented in Mills’ monograph [44], the samples were annealed. On the other hand, our own tests were carried out on a sample without preliminary thermal treatment. This finding may also be confirmed by the fact that the diffusivity differs in the temperature range of 700–800 °C, which, according to the literature [40], may be caused by the δ phase precipitation phenomenon.

**Figure 8:**
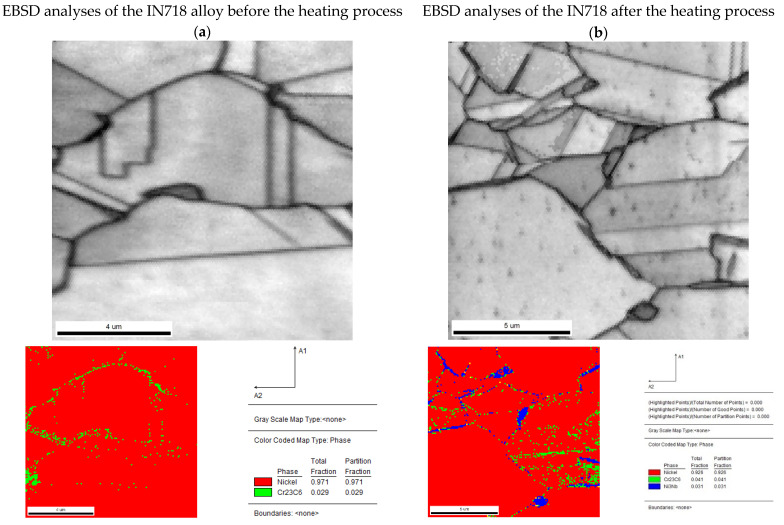
Identification by EBSD microdiffraction of carbide precipitates in the IN718 alloy before the heating process (**a**) and carbide precipitates and the Ni3Nb phase after the heating process (**b**).

This assumption was confirmed by microscopic observations and chemical composition analysis (Figure 6 and Figure 7). In the delivered condition, before the heating process, the structure of the material consists of Ni (Al)-γ solid solution grains with numerous precipitates of M_23_C_6_ carbides located in the boundary areas. X-ray phase analysis also allowed us to identify in the alloy structure the hexagonal Laves phase with a network corresponding to the C_14_ structure of the TiCr_2_ phase. In the material as delivered, no cuboidal precipitates of the γ′ superstructure were observed during the microscopic observations. This was also confirmed by the XRD phase analysis, where no reflections from the crystallographic planes (100) and (110), characteristic for the Ni_3_Al superstructure, were observed on the diffractograms (Figure 6). The heating process carried out to the temperature of 1000 °C, in accordance with the literature data [40], resulted in numerous precipitations of the δ (Ni_3_Nb) phase being observed in the granular matrix of the tested material, without causing other significant changes in the structural structure of the tested material (Figure 7). The heating process carried out to the temperature of 1000 °C, in accordance with the literature data [40], resulted in the formation of precipitates with a noticeably increased content of niobium and nickel (Table 2). This observation, in conjunction with the XRD analysis, allowed us to state that the precipitations of the δ (Ni_3_Nb) phase were observed in the granular matrix of the tested material, without causing any other significant changes in the matrix of the solid solution and carbide precipitates. The final confirmation of the correctness of the identification of the observed precipitates, and in particular, of the formation of the Ni_3_Nb phase, are the results of EBSD microdiffraction, clearly indicating the release of the δ phase under the applied measurement conditions (Figure 8).

## 5. Conclusions

The advantages of the method for determining the thermal diffusivity by the MPM method described in this paper are:

- A significant advantage of the MPM method is the use of the results of measuring the temperature difference $\Delta \Theta \left(t\right)=\Theta_{1}\left(t\right)-\Theta_{2}\left(t\right)$ between the extreme surfaces of the test sample, after a laser shot in its face, to determine the characteristic time τ, and thus the thermal diffusivity $a\left(T_{i}\right)$.
- The use of this method of measuring $\Delta \Theta \left(t\right)$, and thus ${\Delta E}_{th}\left(t\right)$ and $\Delta E\left(t\right)$, allows one to eliminate even components from these measurement signals (*n* = 2, 4, 6,...).
- Therefore, there is a significant expansion of the time interval in which we can treat the logarithm of the function ${\Delta \Theta }_{n=1}\left(t\right)$, ${\Delta E}_{th,n=1}\left(t\right)$, and ${\Delta E}_{n=1}\left(t\right)$ as linearly dependent on;
- A relatively small error in determining the thermal diffusivity by the MPM method, which depends mainly on the accuracy of the measurements of the thickness l of the tested sample, and the characteristic time τ. In this case, the error was estimated to be less than 3%;
- Precise determination of the discrete temperature value $T_{i}=T_{0}+\Theta_{\infty }$ in which $a\left(T_{i}\right)$ is measured as well as averaging the temperature range of this value ${\left.a\left(T\right)\right|}_{T_{i}-0.5\Delta T}^{T_{i}+0.5\Delta T}$; where $\Delta T=4\Theta_{\infty }\exp\left[-t_{2}/\tau \right]$ (see Figure 1a);
- A simple way to determine the ratio of temperature differences $\Delta \Theta \left(t_{1}\right)/\Delta \Theta \left(t_{2}\right)$ between the extreme surfaces of the tested sample by replacing it with $\Delta E\left(t_{1}\right)/\Delta E\left(t_{2}\right)$. At the same time, the values of $\Delta E\left(t_{1}\right)$ and $\Delta E\left(t_{2}\right)$ are taken from the experimentally recorded changes of $\Delta E\left(t\right)$, as shown in Figure 4. Additionally, for small, several-degree changes in temperature $\Delta \Theta \left(t\right)$ in the time interval between $t_{1}$ and $t_{2}$ depending on $\Delta E_{th}\left(t\right)=k_{1}\Delta \Theta \left(t\right)$, it can be assumed that $k_{1}=const$;
- Due to the differential measurement of the thermal Emf in the “CuNi–sample–CuNi” system, the harmful influence of external electromagnetic fields on the measurement signal was eliminated.

Further verification of the MPM method is planned, with particular attention being paid to the application of this method to the study of the thermal diffusivity of materials operating at high temperatures.

## Figures and Tables

**Table 1:** Chemical composition of the tested Inconel 718 together with the chemical compositions of samples from the same material, the results of which are available in the available literature for the thermal diffusivity tests.

	Element [wt.%]										
	Ni	Fe	Cr	Ti	Al	Nb	Mo	Co	Mn	Si	Cu
Investigated sample	50.77	18.25	18.28	1.03	0.83	5.69	2.83	1.22	0.37	0.81	-
Mills [44]	52.5	16.7	19.0	0.9	0.5	5.2	3.1	1.0	0.35	0.35	0.3
MPDB [45]	50–55	16–20	17–21	0.65–1.15	0.2–0.8	4.75–5.5 *^)^	2.8–3.3	max 1.0	max 0.35	max 0.35	max 0.3

*^)^ Nb + Tl.

**Table 2:** Chemical composition of the material (weight %) in the areas marked in Figure 6.

	Area 1	Area 2	Area 3	Area 4	Area 5
C	---	00.87	---	01.02	---
Al	00.52	00.42	00.47	00.41	---
Nb	04.49	11.35	04.22	09.81	15.38
Mo	03.01	02.96	03.00	02.64	00.06
Ti	00.83	01.39	00.83	01.41	01.79
Cr	18.00	12.56	17.78	13.72	10.10
Fe	17.84	12.39	17.88	14.02	10.58
Ni	55.31	58.06	54.87	56.97	62.10

## Data Availability

Data are contained within the article.

## References

[1] Meola C., Carlomagno G.M. (2004). Recent advances in the use of infrared thermography. Meas. Sci. Technol..

[2] Erdoğdu F. (2006). A review on simultaneous determination of thermal diffusivity and heat transfer coefficient. J. Food Eng..

[3] Huang L., Liu L.-S. (2009). Simultaneous determination of thermal conductivity and thermal diffusivity of food and agricultural materials using a transient plane-source method. J. Food Eng..

[4] Hadi S., Nishitani M., Wijayanta A.T., Kurata K., Takamatsu H. (2012). Measurement of Thermal Conductivity and Thermal Diffusivity of Solid Materials Using a Novel Stamp Sensor: A Feasibility Study with Numerical Analysis. J. Therm. Sci. Technol..

[5] San Martin C., Torres C., Esparza D., Bonilla D. (2012). Thermal diffusivity measurements of spherical samples using active infrared thermography. Infrared Phys. Technol..

[6] Cernuschi F., Bison P.G., Marinetti S., Figari A., Lorenzoni L., Grinzato E. Comparison of thermal diffusivity measurement techniques. Proceedings of the 6th International Conference on Quantitative InfraRed Thermography.

[7] Cape J.A., Lehman G.W. (1963). Temperature and Finite Pulse-Time Effects in the Flash Method for Measuring Thermal Diffusivity. J. Appl. Phys..

[8] Glavina M.Y., Scala K.D., Ansorena R., Valle C.E. (2006). Estimation of thermal diffusivity of foods using transfer functions. LWT-Food Sci. Technol..

[9] Baba T., Ono A. (2001). Improvement of the laser flash method to reduce uncertainty in thermal diffusivity measurements. Meas. Sci. Technol..

[10] Maglic K.D., Cezairliyan A., Peletsky V.E. (1992). Compendium of Thermophysical Property Measurement Methods—Volume 2: Recommended Measurement Techniques and Practices.

[11] Vălu O.S., Staicu D., Beneš O., Konings R.J.M., Lajarge P. (2014). Heat capacity, thermal conductivity and thermal diffusivity of uranium–americium mixed oxides. J. Alloys Compd..

[12] Reza A., Zayachuk Y., Yu H., Hofmann F. (2020). Transient grating spectroscopy of thermal diffusivity degradation in deuterium implanted tungsten. Scr. Mater..

[13] Bellucci S., Bovesecchi G., Cataldo A., Coppa P., Corasaniti S., Potenza M. (2019). Transmittance and Reflectance Effects during Thermal Diffusivity Measurements of GNP Samples with the Flash Method. Materials.

[14] Taylor R.E., Magliĉ K.D., Magliĉ K.D. (1984). Compendium of Thermophysical Property Measurement Method.

[15] Vozár L., Hohenauer W. Flash method of measuring the thermal diffusivity, a review. Proceedings of the 16th European Conference on Thermophysical Properties.

[16] Hemberger F., Ebert H.P., Fricke J. (2007). Determination of the Local Thermal Diffusivity of Inhomogeneous Samples by a Modified Laser-Flash Method. Int. J. Thermophys..

[17] Laskar J.M., Bagavathiappan S., Sardar M., Jayakumar T., Philip J., Raj B. (2008). Measurement of thermal diffusivity of solids using infrared thermography. Mater. Lett..

[18] Martínez K., Marín E., Glorieux C., Lara-Bernal A., Calderón A., Peña Rodríguez G., Ivanov R. (2015). Thermal diffusivity measurements in solids by photothermal infrared radiometry: Influence of convection–radiation heat losses. Int. J. Therm. Sci..

[19] Massard H., Pinto C.S.C., Couto P., Orlande H.R.B., Cotta R.M. Analysis of flash method physical models for the measurement of thermal diffusivity of solid materials. Proceedings of the 10th Brazilian Congress of Thermal Sciences and Engineering—ENCIT 2004.

[20] Woodfield P.L., Monde M., Mitsutake Y. (2007). On estimating thermal diffusivity using analytical inverse solution for unsteady one-dimensional heat conduction. Int. J. Heat Mass Trans..

[21] Parker W.J., Jenkins R.J., Butler C.P., Abbot G.L. (1961). Flash Method of Determining Thermal Diffusivity, Heat Capacity, and Thermal Conductivity. J. Appl. Phys..

[22] Ukrainczyk N. (2009). Thermal diffusivity estimation using numerical inverse solution for 1D heat conduction. Int. J. Heat Mass Trans..

[23] Terpiłowski J., Rudzki R., Szczepaniak R., Woroniak G. (2016). Thermal diffusivity investigation of Fe61Ni39, Fe52Ni48 and Fe40Ni60 binary iron–nickel alloys using the modified pulse method. J. Alloys Compd..

[24] Terpiłowski J., Rudzki R., Szczepaniak R., Woroniak G. (2018). Investigation of thermal diffusivity of Fe80Ni20 alloy by means of modified pulse method. J. Alloys Compd..

[25] Hu W., Huang Z., Yu Q., Wang Y., Jiao Y., Lei C., Cai L., Zhai H., Zhou Y. (2020). Ti_2_AlC triggered in-situ ultrafine TiC/Inconel 718 composites: Microstructure and enhanced properties. J. Mater. Sci. Technol..

[26] Chamanfar A., Sarrat L., Jahazi M., Asadi M., Weck A., Koul A.K. (2013). Microstructural characteristics of forged and heat treated Inconel-718 disks. Mater. Des. (1980–2015).

[27] Yang H., Yang J., Huang W., Jing G., Wang Z., Zeng X. (2019). Controllable in-situ aging during selective laser melting: Stepwise precipitation of multiple strengthening phases in Inconel 718 alloy. J. Mater. Sci. Technol..

[28] Kang D.S., Koizumi Y., Yamanaka K., Aoyagi K., Bian H., Chiba A. (2018). Significant impact of yttrium microaddition on high temperature tensile properties of Inconel 713C superalloy. Mater. Lett..

[29] Anbarasan N., Gupta B.K., Prakash S., Muthukumar P., Oyyaravelu P., Kumar R.J.F., Jerome S. (2018). Effect of Heat Treatment on the Microstructure and Mechanical Properties of Inconel 718. Mater. Today Proc..

[30] Karthik G.M., Asghari-Rad P., Sathiyamoorthi P., Zargaran A., Kim E.S., Kim T.S., Kim H.S. (2021). Architectured multi-metal CoCrFeMnNi-Inconel 718 lamellar composite by high-pressure torsion. Scr. Mater..

[31] Li Y., Zhang Z., Guan Y. (2020). Thermodynamics analysis and rapid solidification of laser polished Inconel 718 by selective laser melting. Appl. Surf. Sci..

[32] Rifat M., DeMeter E.C., Basu S. (2020). Microstructure evolution during indentation of Inconel-718 created by additive manufacturing. Mater. Sci. Eng. A.

[33] Zhang H., Li C., Guo Q., Ma Z., Li H., Liu Y. (2019). Improving creep resistance of nickel-based superalloy Inconel 718 by tailoring gamma double prime variants. Scr. Mater..

[34] Wan H.Y., Luo Y.W., Zhang B., Song Z.M., Wang L.Y., Zhou Z.J., Li C.P., Chen G.F., Zhang G.P. (2020). Effects of surface roughness and build thickness on fatigue properties of selective laser melted Inconel 718 at 650 °C. Int. J. Fatigue.

[35] Parida R.P., Senthilkumar V. (2021). Experimental studies of defect generation in selective laser melted Inconel 718 alloy. Mater. Today Proc..

[36] Ruan J.J., Ueshima N., Oikawa K. (2020). Growth behavior of the δ-Ni3Nb phase in superalloy 718 and modified KJMA modeling for the transformation-time-temperature diagram. J. Alloys Compd..

[37] Zhao Y., Aoyagi K., Daino Y., Yamanaka K., Chiba A. (2020). Significance of powder feedstock characteristics in defect suppression of additively manufactured Inconel 718. Addit. Manuf..

[38] Wang K., Liu Y., Sun Z., Lin J., Lv Y., Xu B. (2020). Microstructural evolution and mechanical properties of Inconel 718 superalloy thin wall fabricated by pulsed plasma arc additive manufacturing. J. Alloys Compd..

[39] Jambor M., Bokuvka O., Novy F., Trsko L., Belan J. (2017). Phase Transformations in Nickel base Superalloy Inconel 718 during Cyclic Loading at High Temperature. Prod. Eng. Arch..

[40] Zhao Y., Hao L., Zhang Q., Xiong W. (2022). Phase transformations during continuous cooling in Inconel 718 alloys manufactured by laser powder bed fusion and suction casting. Mater. Charact..

[41] Terpiłowski J., Szczepaniak R., Woroniak G., Rudzki R. (2013). Adaptation of the modified pulse method for determination of thermal diffusivity of solids in the vicinity of the second-order phase transition point. Arch. Thermodyn..

[42] Terpiłowski J., Jóźwiak S., Rudzki R., Szczepaniak R., Woroniak G. (2020). Investigation of Phase Transformation of Fe65Ni35 Alloy by the Modified Pulse Method. Materials.

[43] Terpiłowski J. (2003). A modified flash method for determination of thermal diffusivity in solids. Arch. Thermodyn..

[44] Mills Kenneth C. (2002). Recommended Values of Thermophysical Properties for Selected Commercial Alloys.

[45] (2012). Material Property Database. MPDB; v. 7.49; JAHM Software 1 nc.

